# Transcriptomic profiling of long non-coding RNAs in dermatomyositis by microarray analysis

**DOI:** 10.1038/srep32818

**Published:** 2016-09-08

**Authors:** Qing-Lin Peng, Ya-Mei Zhang, Han-Bo Yang, Xiao-Ming Shu, Xin Lu, Guo-Chun Wang

**Affiliations:** 1 Department of Rheumatology, China-Japan Friendship Hospital, Beijing, China

## Abstract

Long non-coding RNAs (lncRNAs) are prevalently transcribed in the genome and have been found to be of functional importance. However, the potential roles of lncRNAs in dermatomyositis (DM) remain unknown. In this study, a lncRNA + mRNA microarray analysis was performed to profile lncRNAs and mRNAs from 15 treatment-naive DM patients and 5 healthy controls. We revealed a total of 1198 lncRNAs (322 up-regulated and 876 down-regulated) and 1213 mRNAs (665 up-regulated and 548 down-regulated) were significantly differentially expressed in DM patients compared with the healthy controls (fold change>2, *P* < 0.05). Subgrouping DM patients according to the presence of interstitial lung disease and anti-Jo-1 antibody revealed different expression patterns of the lncRNAs. Pathway and gene ontology analysis for the differentially expressed mRNAs confirmed that type 1 interferon signaling was the most significantly dysregulated pathway in all DM subgroups. In addition, distinct pathways that uniquely associated with DM subgroup were also identified. Bioinformatics prediction suggested that linc-DGCR6-1 may be a lncRNA that regulates type 1 interferon-inducible gene USP18, which was found highly expressed in the perifascicular areas of the muscle fibers of DM patients. Our findings provide an overview of aberrantly expressed lncRNAs in DM muscle and further broaden the understanding of DM pathogenesis.

Dermatomyositis (DM) is an autoimmune disease of unknown etiopathogenesis characterized by symmetrical skeletal muscle weakness and skin rashes^1^,^2^. Clinical heterogeneity is a significant feature of DM patients. In particular, a multitude of DM patients are complicated with interstitial lung disease (ILD) and anti-Jo-1 antibody appears in the serum of many patients, while patients without lung involvement and serum myositis specific autoantibodies are also commonly seen. The mechanism that causes this obvious clinical heterogeneity is not clear. Recent studies have shown that dysregulated gene expression of particular molecules may play significant roles in DM disease development. As an example, up-regulation of MHC-I in skeletal muscle successfully induced self-sustaining autoimmune myositis in a mouse model, providing strong evidence that MHC-I significantly contributes to the pathogenesis of myositis^3^. Additionally, studies have revealed that a number of coding genes were differentially expressed in myositis muscle tissue^4^,^5^,^6^.

As we know, the vast majority of human genome was considered to be noncoding genes^7^. The noncoding RNAs are a large group of transcriptional outputs without protein-coding function and can be divided into two major groups, short noncoding RNA and large noncoding RNA. A growing body of evidence indicate that noncoding RNAs play an important role in regulating multiple processes of gene expression. In contrast with microRNAs which are well studied short noncoding RNAs that have regulatory functions, the long noncoding RNAs (lncRNAs) are recently annotated and remain to be fully understood. LncRNAs are RNA transcripts longer than 200 nucleotides with no coding potential^8^,^9^. Like messenger RNAs, they are mostly transcribed by RNA polymerase II, but they do not undergo the subsequent mRNA processing steps. The regulatory functions of lncRNAs have been increasingly revealed. Studies have found that regulation by lncRNAs can affect mRNA transcription, splicing, export, stability, and translation^10^.

Muscle damage is a significant characteristic of myositis patients. Dysregulation of gene expression may probably participate in the process of muscle damage in myositis, while limited information is known about the regulatory factors. Eisenberg *et al.* firstly reported the altered expression of several microRNA molecules in the muscle tissue of myositis patients^11^. Further investigation demonstrated that microRNA-1, -133, -206, -126 contributed to the pathogenesis of myositis as important regulating factors of relevant gene expression^12^,^13^. Despite emerging data showing the regulatory role of microRNAs in myositis, there is a paucity of information concerning the expression and potential role of lncRNAs in myositis.

High throughput genome screening can provide a panoramic view of gene expression in pathological conditions and may consequently provide new insights for disease pathomechanism. Therefore, in order to explore the expression pattern of lncRNAs in different DM clinical subtypes and to find potential regulatory lncRNAs in DM muscle, we profiled the lncRNAs and mRNAs in muscle tissue by using microarray analysis.

## Results

### Expression profiles of lncRNAs and mRNAs in DM muscle

**Figure 1:**
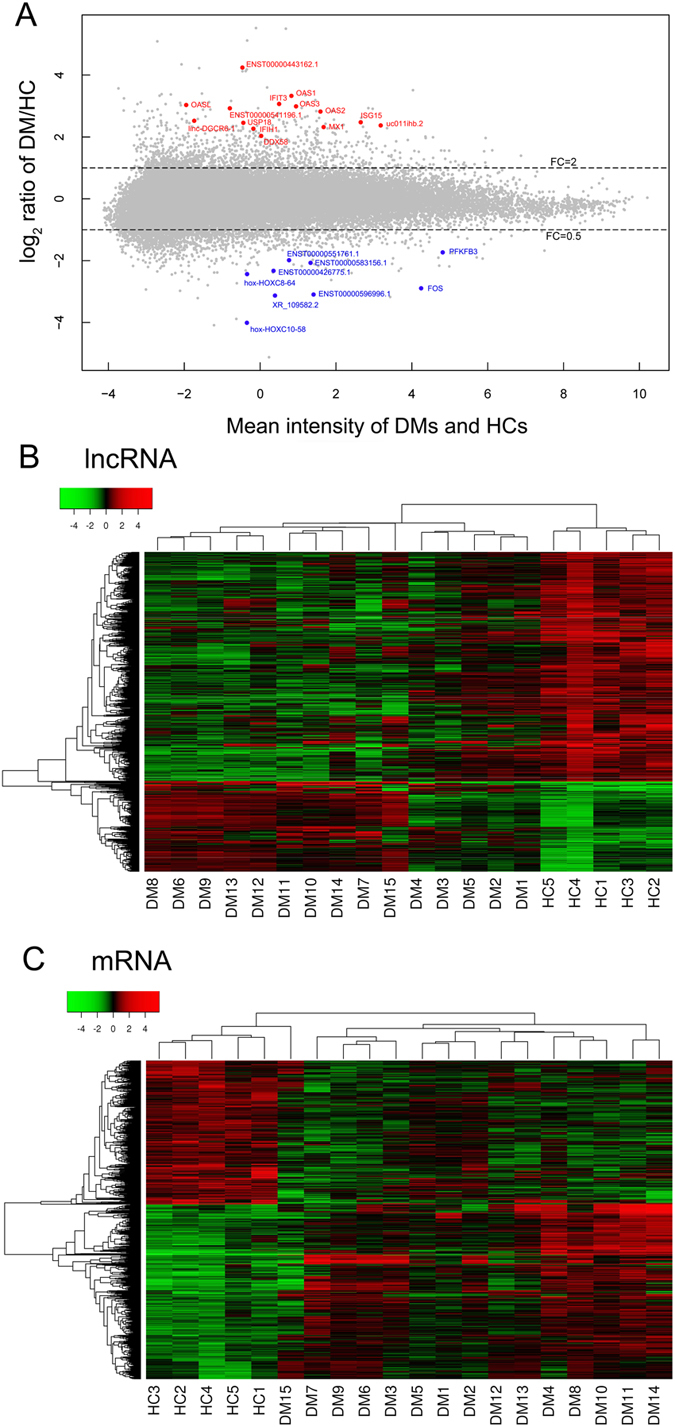
Expression profiles of lncRNAs and mRNAs in dermatomyositis(DM) patients and healthy controls (HC) by microarray analysis. The MA plot gives a quick overview of the distribution of the microarray gene expression data (**A**). The values presented in the MA plot are averaged normalized values (log_2_-scaled). The lncRNAs and mRNAs above the top dashed line and below the bottom dashed line are those with a >2-fold or <0.5-fold change in expression between DMs and HCs. In addition, several lncRNAs and mRNAs that mentioned in this study have been highlighted in the MA plot. Heat map and hierarchical clustering are presented to show the variation in lncRNA (**B**) and mRNA (**C**) expression between DMs and HCs. Green strip indicates high relative expression and red strip indicates low relative expression.

Through microarray analysis, we examined the lncRNA and mRNA expression profiles in the muscle of 15 DM patients and 5 healthy controls. The MA plot is shown to reflect the overall data quality (Fig. 1A). Hierarchical clustering was performed based on the lncRNA and mRNA expression values in the microarray (Fig. 1B,C). The microarray data revealed 1213 mRNAs and 1198 lncRNAs were differentially expressed in DM patients compared with the control group. Among them, 665 mRNAs were upregulated, and 548 mRNAs were downregulated in DM patients. Regarding the lncRNAs, we found that 322 were upregulated and 876 were downregulated in DM patients. Table 1 shows the top 10 upregulated and downregulated lncRNAs. All lncRNAs and mRNAs that were aberrantly expressed with an absolute fold-change greater than 5 are listed in Supplementary datasheet 2.

**Figure 2:**
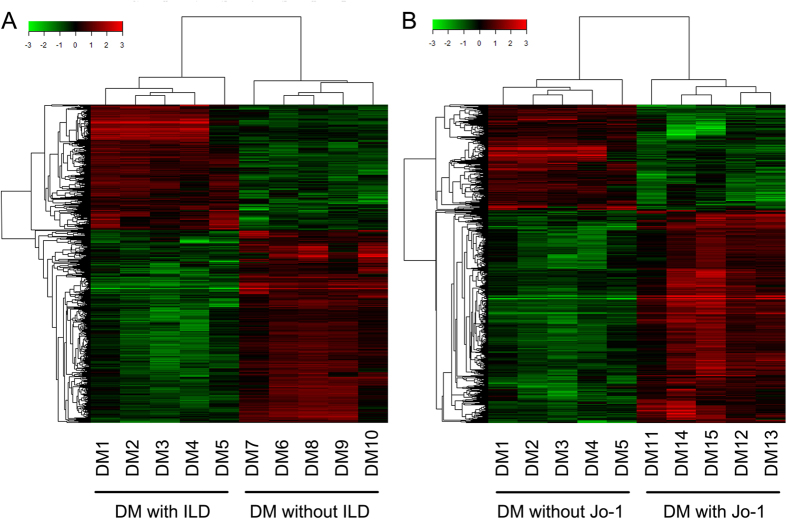
Heat map and hierarchical clustering showing differential expression pattern of lncRNAs in dermatomyositis patients subgrouping according to the presence of interstitial lung disease (ILD) (**A**) and anti-Jo-1 autoantibody (**B**).

Additionally, we subgrouped the DM patients according to the presence of ILD and found differentially expressed lncRNAs in DM patients with ILD compared to patients without ILD. Figure 2A shows the different expression patterns of lncRNAs in the two subgroups. Additionally, the top 10 upregulated and downregulated lncRNAs are listed in Supplementary Table S3, with ENST00000428205.1 and ENST00000450016.1 as the lncRNAs that display the most significant increase and decrease, respectively. Moreover, we also identified certain lncRNAs that were expressed differently between DM patients with anti-Jo-1 antibody and DM patients without the antibody (Fig. 2B), among which we found that ENST00000587036.1 and XR_110948.1 expressions differed the most between the two subgroups (Supplementary Table S4).

### Quantitative real-time PCR validation

**Figure 3:**
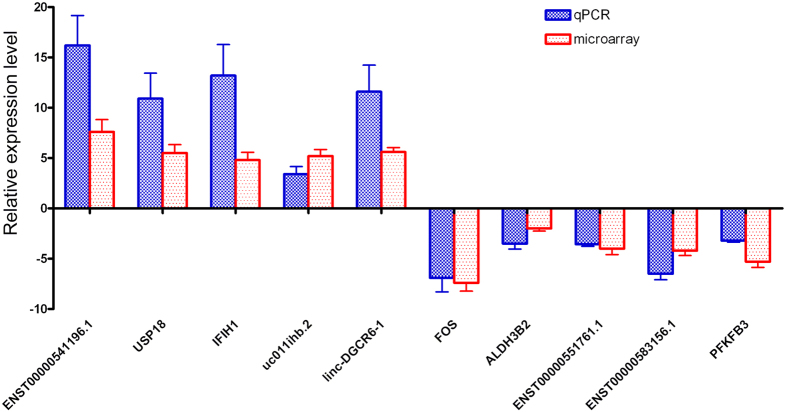
Comparison between the results of microarray analysis and quantitative PCR assay. Five lncRNAs (ENST00000541196.1, uc011ihb.2, linc-DGCR6-1, ENST00000551761.1, ENST00000583156.1) and five mRNAs (USP18, IFIH1, FOS, ALDH3B2, PFKFB3) were selected to be analyzed by quantitative real-time PCR to validate their expression levels relative to healthy controls. The results indicated that the microarray results correlated well with the qPCR data.

Five lncRNAs and five mRNAs were selected to be analyzed by quantitative RT-PCR to validate their expression levels in the 15 DM patients involved in microarray analysis. The qRT-PCR results showed that the expression levels of lncRNAs- ENST00000541196.1, uc011ihb.2, linc-DGCR6-1, and of mRNAs- USP18, IFIH1 were significantly increased (P values all < 0.05) in DM patients compared to that in healthy controls. In addition, the expression of lncRNAs- ENST00000551761.1, ENST00000583156.1 and of mRNAs- FOS, ALDH3B2, PFKFB3 were significantly decreased (P values all < 0.05). The qRT-PCR results were consistent with the results of the microarray analysis (Fig. 3), and thus providing reliable validation for the microarray results.

### lncRNA classification and subgroup analysis

According to previous reports, lncRNAs can be divided to three main subgroups: HOX lncRNAs^14^, lncRNAs with enhancer-like function^15^, and large intergenic noncoding RNAs (lincRNAs)^16^. Our profiling data indicated that the differentially expressed lncRNAs consisted of 110 lncRNAs with enhancer-like function, 5 lincRNAs, and no HOX lncRNAs. Supplementary datasheet 3 shows all of these lncRNA subsets.

### Bioinformatics analysis of differentially expressed mRNAs in DM patients

**Figure 4:**
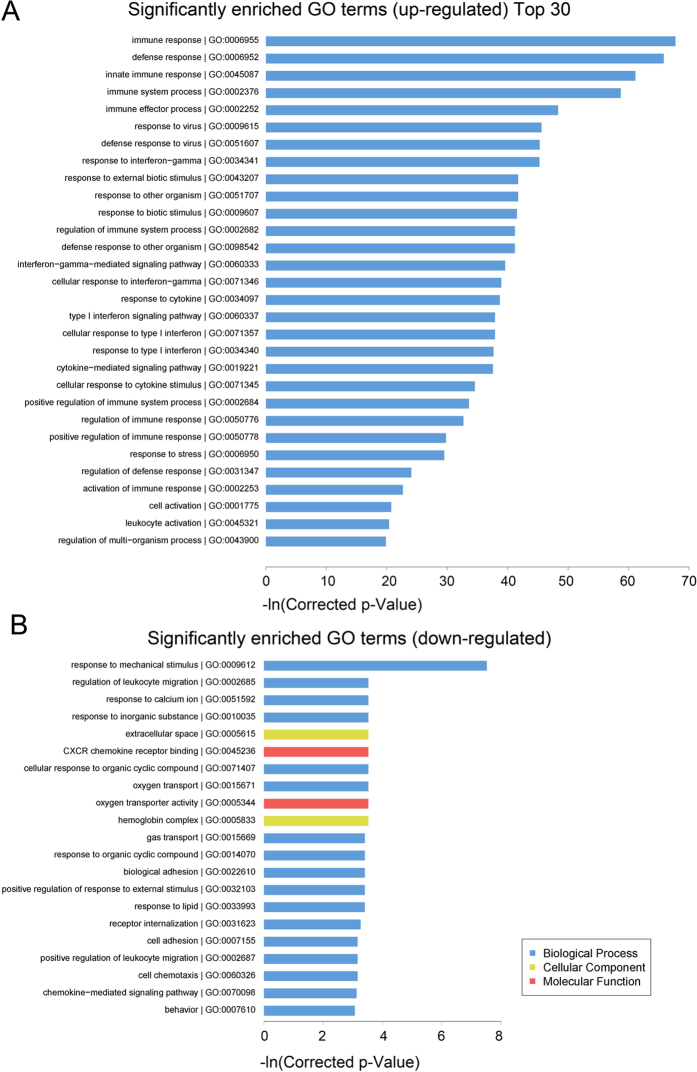
Gene Ontology (GO) analysis of differentially expressed mRNAs in DM patients. The value of -ln (p value) was calculated to reflect the significance of GO term enrichment. The top 30 enriched GO terms of up-regulated mRNAs (**A**) and down-regulated mRNAs (**B**) are shown.

Gene Ontology (GO) is a bioinformatics initiative that seeks to better represent gene and gene product attributes, providing three structured networks of defined terms: biological process, cellular component, and molecular function. By applying GO analysis, we found that the genes corresponding to the up-regulated mRNA transcripts in DM were involved in 306 GO terms in the biological process network, 34 GO terms in the cellular component network and 11 GO terms in the molecular function network. Additionally, the genes corresponding to the down-regulated mRNAs were found to be involved in 17 GO terms in biological process, 2 GO terms in cellular component, 2 GO terms in molecular function. The top 30 significant GO terms associated with dysregulated mRNAs are shown in Fig. 4. Notably, GO clustering revealed that “type 1 interferon-mediated signaling pathway” (GO:0060337), “response to type 1 interferon” (GO:0034340) were significantly up-regulated, indicating that dysregulated type 1 interferon signaling play a role in DM pathogenesis.

**Figure 5:**
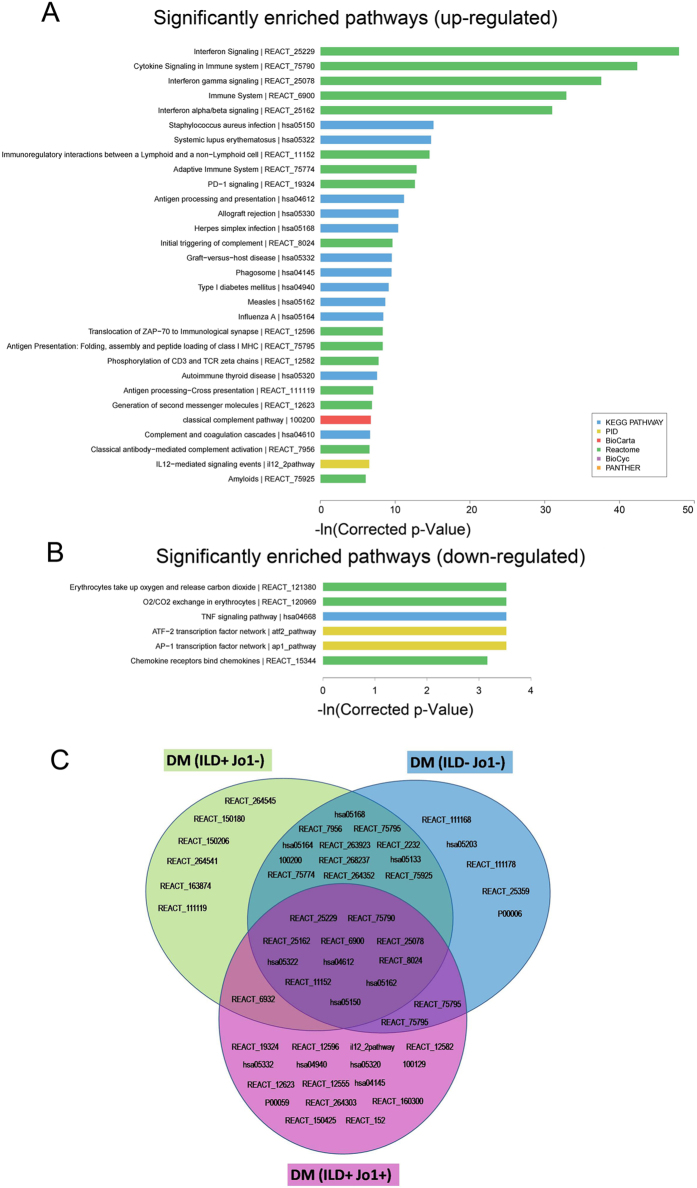
Pathway analysis of the differentially expressed mRNAs. (**A**) The 30 most significant pathways of up-regulated mRNAs. (**B**) The significant pathways of the down-regulated mRNAs. Enrichment score values were calculated as -ln (p values). (**C**) Distinct and shared pathways in DM subgroups “ILD+ Jo1−”, “ILD− Jo1−”, “ILD+ Jo1+”. The detailed information of the pathways involved in the figure is presented in Supplementary datasheet 4.

We further performed pathway analysis on the differentially expressed mRNAs according to the following databases: the latest version of Kyoto Encyclopedia of Genes and Genomes (KEGG) database (http://www.genome.jp/kegg), PID (http://pid.nci.nih.gov/), BioCyc (http://biocys.org/), Reactome (http://www.reactome.org/), and Panther (http://www.pantherdb.org/). Consequently, the biological pathways that were significantly enriched with these differentially expressed mRNAs were uncovered. The results showed that a total of 74 pathways were associated with the up-regulated mRNA transcripts, with “Interferon Signaling” recognized as the most enriched network. Furthermore, we also found 6 pathways that were significantly related to down-regulated mRNAs, “Erythrocytes take up oxygen and release carbon dioxide” being the most enriched one. The top 30 enriched pathways of up-regulated mRNAs and down-regulated mRNAs are shown in Fig. 5A,B.

Moreover, we divided the DM patients into three subgroups: “ILD+ Jo1−”, “ILD− Jo1−”, “ILD+ Jo1+” according to the presence/absence of ILD and anti-Jo-1 antibody. Then, we identified top 30 dysregulated pathways in each subgroup compared to healthy controls. We analyzed distinct pathways and shared pathways in these three DM subgroups, which are shown in Fig. 5C. Specifically, pathways including Interferon Signaling (REACT_25229), Cytokine Signaling in Immune System (REACT_75790), Interferon Alpha/Beta Signaling (REACT_25162) are recognized as shared pathways that are dysregulated in all the three subgroups. We also found pathways uniquely associated with DM subgroup “ILD+ Jo1−” such as RMTs methylate histone arginines (REACT_264545). Moreover, the pathways Endosomal/Vacuolar pathway (REACT_111168) and PD-1 signaling (REACT_19324) were found to be related with DM subgroups “ILD− Jo1−”, “ILD+ Jo1+” respectively.

### Construction of the mRNA-lncRNA co-expression network

Based on the results of correlation analyses of the differentially expressed lncRNAs and mRNAs, mRNA-lncRNA co-expression networks were built. In total, we identified 718 pairs of co-expressed lncRNA-mRNA (Supplementary datasheet 5). Subsequently, 122 co-expression networks were constructed. Supplementary Figure S1 shows a representative mRNA-lncRNA co-expression network which involves 4 lncRNAs and 20 mRNAs.

### Target gene prediction for the differentially expressed lncRNAs

According to the mRNA-lncRNA co-expression networks, we predicted the coding genes that would be targeted by the differentially expressed lncRNAs by using bioinformatical approach. The prediction identified 12 lncRNA-mRNA pairs (Supplementary Table S5) and suggested the likelihood of these lncRNAs as regulators of corresponding mRNA expression. Of the highest interest, we noticed a lncRNA named linc-DGCR6-1 may target USP18, which is known as a type 1 interferon-inducible protein and considered to be a key regulator of interferon signaling^17^,^18^.

### High expression levels of USP18 in the muscles of DM patients

**Figure 6:**
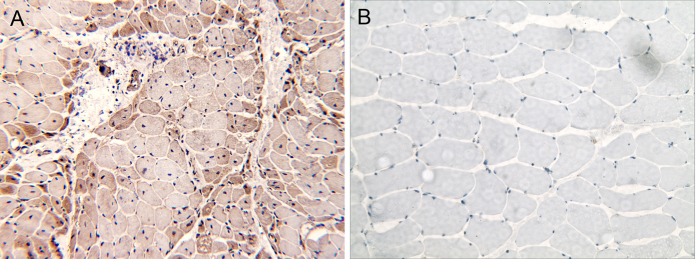
Immunohistochemistry staining of USP18 in the muscle of dermatomyositis patients. Muscle biopsies from a representative DM patient showed significant USP18 staining in perifascicular myofibers (**A**), while no obvious USP18 expression was observed in healthy control (**B**).

In order to validate the microarray findings of increased expression of USP18 in DM, we analyzed the expression of the USP18 protein by immunohistochemistry staining. Obvious expression of USP18 was prominently found in the perifascicular areas of the muscle fibers of DM patients. In contrast, we found no significant USP18 expression in healthy control muscle samples (Fig. 6).

## Discussion

In this study, we profiled the expression of lncRNAs and mRNAs in DM muscle tissue by microarray analysis and identified lncRNAs and mRNAs that were differentially expressed in DM patients. Stratification by clinical subgroup according to the presence of ILD and of the anti-Jo-1 autoantibody revealed different expression patterns of lncRNAs in DM subgroups. Unique and shared pathways related to the three DM subsets were also unveiled. In particular, type 1 interferon signaling was found to be the most significantly dysregulated pathway in all DM subgroups, indicating that type 1 interferon may play a role in DM pathogenesis. Through constructing mRNA-lncRNA co-expression networks, we predicted the target genes of the differentially expressed lncRNAs by using a bioinformatic approach. Of note, we identified a lncRNA that is likely involved in the regulation of type 1 interferon-inducible molecule USP18. Additionally, immunohistochemistry staining showed that upregulated expression of USP18 proteins were mainly observed in perifascicular atrophy myofibers of DM patients.

In contrast to the previous consideration of noncoding transcripts as junk DNA with no functional purpose^19^, rapidly expanded research in the past decade has demonstrated that non-coding RNAs are essential for a variety of cellular processes^20^. The regulatory roles of non-coding RNAs have attracted considerable attention in recent years. In particular, recent findings have demonstrated that lncRNAs play significant roles in the regulation of immune functions^21^,^22^,^23^,^24^. Moreover, a growing body of evidence suggested that lncRNAs dysregulation may play a vital role in autoimmune diseases including systemic lupus erythematosus, rheumatoid arthritis^24^. Intriguingly, emerging data have unveiled lncRNAs that are involved in myopathies and that may play a role in muscle differentiation, regeneration and function^10^,^25^,^26^,^27^. However, little information is known about lncRNAs in DM. Systemic genome screening is a powerful tool for identifying differentially expressed RNA transcripts, which has been widely used to examine the expression profile of lncRNAs in various diseases^28^,^29^,^30^,^31^,^32^,^33^. Thereby we sought to investigate the expression profile of lncRNAs in DM muscle and relate it to the mRNA expression profile by using high throughput microarray analysis.

To our knowledge, this is the first study that address the transcriptomic profiling of lncRNAs in DM patients. In addition to a large number of differentially expressed mRNAs, we also found thousands of lncRNAs differentially expressed in DM muscle tissue. Our further bioinformatics prediction analysis points to a set of lncRNAs that may be potential gene regulators in DM pathogenesis. Therefore, the present data will unveil intriguing areas of inquiry for more in-depth exploration.

In order to gain insights into the biological pathways potentially involved in DM, pathway analysis was performed. The results revealed that several pathways that are involved in other autoimmune disorders - such as systemic lupus erythematosus, type 1 diabetes mellitus, autoimmune thyroid disease - were significantly dysregulated in DM, which suggests that DM may share some pathways with other autoimmune diseases. Recent genetic studies demonstrated that DM has a shared genetic etiology with other autoimmune disorders^34^,^35^. Our finding of several pathways that contribute to other autoimmune diseases were also dysregulated in DM, therefore, indicates that, besides genetic overlap, molecular pathways may also be shared by DM and other autoimmune disorders.

In our study, both GO and pathway analysis results suggested a role of type 1 interferon in DM, which is in accordance with previous reports^6^. An increasing number of investigations have shown highly expression of type 1 interferon-inducible proteins in the muscle and skin tissue of DM patients^4^,^5^,^6^,^36^,^37^. It is supposed that the overexpression and intracellular accumulation of type 1 interferon-inducible proteins might result in cellular toxicity in muscle fibers^38^. Moreover, the recent identification of an autoantibody against IFIH1(also known as anti-MDA5), which is a classic type 1 interferon-inducible protein in the patients with DM especially clinically amyopathic DM^39^, provides further evidence for the abnormality related to type 1 interferons in DM^40^. These studies imply that type 1 interferon pathway significantly contributes to the pathogenesis of DM. In our study, we also found significantly upregulated transcription levels of type 1 interferon-inducible genes, including IFN-stimulated ubiquitin-like modifier protein (ISG15), USP18, IFIT3, and IFIH1, which is consistent with previous reports^4^,^5^,^6^,^36^. Further analysis via immunohistochemistry staining, we confirmed that USP18 proteins were preferentially overproduced in perifascicular atrophy myofibers. Therefore the expression pattern of USP18 is similar to other type 1 interferon-inducible molecules such as ISG15 and MX1 which were found to be highly expressed in the perifascicular myofibers of DM muscle^36^. Thus, our study provides further evidence indicating the potential role of type 1 interferon signaling in DM pathogenesis.

On the other hand, although type 1 interferon signaling was the most significant shared pathway that was dysregulated in all DM subgroups, distinct pathways were also found in different DM subsets. Recently, Rothwell *et al.* revealed distinct genetic associations between PM, and DM and JDM, suggesting different predominating pathophysiology in different clinical subgroups^41^. Our data indicated that dysregulated molecular pathways may contribute to the clinical heterogeneity of DM patients.

What is more interesting, bioinformatics prediction in our study suggested that linc-DGCR6-1 might be a potential regulator of the USP18 gene, as linc-DGCR6-1 contains overlap sequence with the 3′ UTR of USP18 gene. Together with the finding of a significant correlation between the expression levels of linc-DGCR6-1 and the USP18 mRNA, our results indicate potential regulatory role for linc-DGCR6-1 in the expression of the USP18 gene. Recently, Gomez *et al.* reported that an enhancer-like lncRNA, termed NeST, functions in either *cis* or *trans* to promote interferon-γ expression^42^, providing direct evidence for a regulatory role that lncRNA may play in interferon pathway. Although further investigation is necessary to confirm the details of the role, our study suggests that lncRNAs may probably contribute to DM pathogenesis by regulating the expression of type 1 interferon-inducible molecules.

However, we acknowledge that our study has some limitations. First of all, the sample size of DM patients included in the microarray analysis was relatively small. This was partly due to our strict selection only of patients with very similar clinical features and muscle pathological manifestations. Additionally, the target genes of the differentially expressed lncRNAs were just predicted by bioinformatics approach, which require to be confirmed by biological function analysis.

Taken together, our study describes a molecular overview of aberrantly expressed lncRNAs in DM muscle and provide novel insights into the pathogenesis of DM. Further investigation of lncRNA function in DM may help to expand our understanding of the molecular mechanism of DM muscle damage.

## Methods

### Patients

From the inpatients who visited China-Japan Friendship Hospital, 15 patients who were diagnosed with DM according to Bohan and Peter criteria were recruited for this study. The diagnoses of the 15 DM patients were biopsy-proven and also fulfilled the ENMC criteria^43^. They all had typical skin rash and muscle weakness. Patients were considered to have ILD if they fulfilled the following criteria: (i) restrictive lung function impairment (total lung capacity (TLC), and diffusion capacity for carbon monoxide of the lung (DLCO) <80% of predicted), and (ii) radiographic signs of ILD on HRCT (nodular, reticulonodular, linear or ground-glass opacities; consolidations; irregular interface; honeycombing; or traction bronchiectasis). Females outnumbered males in the cohort (13 females, 2 males). The DM patients ranged from 28–67 years of age. The clinical characteristics of the enrolled subjects are summarized in Supplementary Table S1. Magnetic resonance imaging-directed muscle biopsies were performed for diagnostic purposes, and the muscle tissue samples were stored in liquid nitrogen before use. All 15 DM patients received no treatment prior to biopsy. Control muscle tissue samples were obtained from 5 trauma patients who did not suffer from muscular diseases. This study was performed with the approval of the Human Ethics Board of China-Japan Friendship Hospital (Beijing, China) in accordance with the Declaration of Helsinki for human research. Written informed consent was obtained from all participating individuals.

### RNA extraction

Total RNA containing small RNA was extracted from frozen muscle tissue by using the Trizol reagent (Invitrogen) and purified with mirVana miRNA Isolation Kit (Ambion, Austin, TX, USA) according to manufacturer’s protocol. The purity and concentration of RNA were determined from OD260/280 readings using the NanoDrop ND-1000 spectrophotometer (NanoDrop Technologies, Wilmington, DE, USA). RNA integrity was determined via 1% formaldehyde denaturing gel electrophoresis.

### Fabrication of microarray

The microarray employed in the current study was Agilent human lncRNA + mRNA Array V4.0, which was designed with four identical arrays per slide (4 × 180 K format) with each array containing probes interrogating about 41,000 human lncRNAs and about 34,000 human mRNAs. The lncRNA target sequences were selected from multiple databases including GENCODE/ENSEMBL, Human LincRNA Catalog, RefSeq, UCS, NRED (ncRNA Expression Database), LNCipedia, H-InvDB, LncRNAs-a (Enhancer-like), Antisense ncRNA pipeline, Hox ncRNAs, UCRs, and 848 unpublished lncRNAs from the Chen Runsheng laboratory (Institute of Biophysics, Chinese Academy of Science, Beijing, China). The mRNAs were collected from RefSeq Build 50, Ensemble Release 52, Unigene Build 216, GenBank (April 2009), and Broad Institute TUCP transcripts catalog (Nov 2011) by Agilent, which have also been used in Agilent-039494 SurePrint G3 Human GE v2 8 × 60 K Microarray. The array also contains 4974 Agilent control probes for hybridization quality control.

### Microarray hybridization and computational analysis

Microarray hybridization was performed according to the standard procedure by CapitalBio Corporation, Beijing, China. Briefly, each purified RNA sample was amplified and transcribed into cRNA along the entire length of the transcripts without 3′ bias by using a random priming method. Subsequently, the cRNAs were transcribed into cDNAs and labeled with a fluorescent dye (Cy3-dCTP) by using Labeling Kit (CapitalBio, Beijing, China). The labeled cDNAs were purified by a PCR NucleoSpin Extract II Kit (MN, Germany) and then were hybridized onto a human lncRNA + mRNA Array V4.0. The hybridization was performed in a Agilent Hybridization Oven overnight at a rotation speed of 20 rpm and a temperature of 42 °C and washed with two consecutive solutions (0.2% SDS, 2× saline sodium citrate buffer at 42 °C for 5 min, and 0.2× saline sodium citrate buffer for 5 min at room temperature).

The lncRNA + mRNA array data summarization, normalization and quality control were analyzed by using the GeneSpring software V12.0 (Agilent Technologies). Fold-change greater than 2 or less than 0.5, and a Benjamini-Hochberg corrected p value less than 0.05 were considered as the criteria for differential expressed genes selection. The data was Log_2_ transformed and median-centered by genes using the Adjust Data function of CLUSTER 3.0 software then further analyzed with hierarchical clustering with average linkage. Finally, tree visualization was performed by using Java Treeview (Stanford University School of Medicine, Stanford, CA, USA).

### Quantitative real-time PCR (qRT-PCR) assay

Quantitative real-time PCR analysis was further carried out to validate the expression levels of candidate genes. Two μg of total RNA was reverse transcribed using the GoTaq^®^ 2-Step RT-qPCR system (Promega, Madison, USA) according to the manufacturer’s protocol. Fluorescent quantitative RT-PCR was performed using the ABI PRISM 7500 system (Applied Biosystems, Foster City, USA) according to standard methods. Five differentially expressed mRNAs and five additional lncRNAs were chosen to validate the gene chip results using qRT-PCR. Specific primers of each gene are listed in Supplementary Table S2. The relative fold change was calculated using the 2^−ΔΔCt^ method normalized to GAPDH. All experiments were performed in triplicate.

### Construction of the mRNA-lncRNA co-expression network

The mRNA-lncRNA co-expression network was constructed based on the correlation analysis between the differentially expressed lncRNAs and mRNAs. For each pair of genes, the Pearson correlation analysis was performed and the significantly correlated pairs were chosen to construct the network. LncRNAs and mRNAs with Pearson correlation coefficients 0.95 or greater were selected to construct the network through the software Cytoscape.

### Target gene prediction

The cis-acting lncRNA predictions were based on the tight correlations (Pearson correlation coefficient greater than 0.99) between the lncRNA and a group of expressed protein-coding genes. These lncRNA reside at genomic loci where a protein-coding gene and a lncRNA gene are within 10 kb of each other along the genome. Therefore, “cis” refers to the same locus (not necessarily same-allele) regulatory mechanisms, which include antisense-mediated regulation by the lncRNAs of protein-coding genes that are encoded at the same locus. The trans-predictions were made using blat tools (Standalone BLAT v. 35 × 1 fast sequence search command line tool, downloaded from: http://hgdownload.cse.ucsc.edu/admin/exe/) to compare the full sequence of the lncRNAs with the 3′ UTR of its co-expressed mRNAs, using the default parameter setting.

### Immunohistochemistry staining

Eight-μm-thick unfixed cryostat muscle sections were collected from diagnostic muscle biopsies. Anti-human ubiquitin-specific peptidase 18 (USP18) polyclonal antibodies (Abcam, Cambridge, UK) were used as primary antibodies for detecting USP18 protein at a working concentration of 5 μg/ml. Horseradish peroxidase-conjugated anti-rabbit IgG (Santa Cruz Biotechnology, Santa Cruz, USA) was used as a secondary antibody. Rabbit IgG isotype control (Abcam, Cambridge, UK) was used as a negative control for the primary antibody. The immunohistochemistry staining was performed as previously described^44^.

### Statistical analysis

Statistical analysis was performed using SPSS V.16.0 (SPSS, Chicago, USA). The fold change and the Student’s t-test were used to analyze the statistical significance of the microarray and RT-PCR results. Additionally, the Benjamini-Hochberg FDR (the FDR cutoff was 0.05) was used for multiple-testing correction. P-values < 0.05 (two-tailed) were considered statistically significant.

## Additional Information

**How to cite this article**: Peng, Q.-L. *et al.* Transcriptomic profiling of long non-coding RNAs in dermatomyositis by microarray analysis. *Sci. Rep.* **6**, 32818; doi: 10.1038/srep32818 (2016).

## Supplementary Material

Supplementary Dataset 1

Supplementary Dataset 2

Supplementary Dataset 3

Supplementary Dataset 4

Supplementary Dataset 5

## Figures and Tables

**Table 1:** Top 10 upregulated and downregulated lncRNAs in dermatomyositis patients examined by microarray analysis.

lncRNAs	Source database	Fold change	P value*
Upregulated			
ENST00000584157.1	ENSEMBL	34.04	0.0000069
RNA147334	unpublished data#	20.32	0.0000101
ENST00000443162.1	ENSEMBL	18.92	0.018823
ENST00000450016.1	ENSEMBL	17.45	0.021193
ENST00000599078.1	ENSEMBL	11.85	0.000313
ENST00000467369.1	ENSEMBL	10.55	0.0000765
NR_073063.1	RefSeq	8.72	0.0000594
RNA147023	unpublished data#	8.54	0.0074663
ENST00000478808.2	ENSEMBL	8.36	0.0000697
NR_038996.1	RefSeq	8.36	0.0012395
Downregulated			
hox-HOXC10-58	HOX Loci	16.12	0.0029392
XR_109582.2	RefSeq	8.76	0.0104836
ENST00000596996.1	ENSEMBL	8.56	0.0086601
ENST00000521815.1	ENSEMBL	5.74	0.0004769
ENST00000509150.1	ENSEMBL	5.46	0.0000044
hox-HOXC8-64	HOX Loci	5.41	0.0007549
ENST00000591400.1	ENSEMBL	5.31	0.0003317
RNA147037	unpublished data#	5.13	0.0000159
ENST00000426775.1	ENSEMBL	5.03	0.0235179
ENST00000582108.1	ENSEMBL	4.99	0.0047212

^*^The displayed P values have been adjusted by Benjamini-Hochberg correction.

^#^Unpublished lncRNAs from Chen Runsheng laboratory (Institute of Biophysics, Chinese Academy of Science, Beijing, China).
