# Supplementary material for: Transcriptomic profiling of long non-coding RNAs in dermatomyositis by microarray analysis
Source: Sci Rep. 2016 Sep 8;6:32818. doi: 10.1038/srep32818 (PMC5015085; doi:10.1038/srep32818)
Supplement: Supplementary Dataset 1 [file srep32818-s1.doc]

Transcriptomic profiling of long non-coding RNAs in dermatomyositis by microarray analysis

Qing-Lin Peng, Ya-Mei Zhang, Han-Bo Yang, Xiao-Ming Shu, Xin Lu, Guo-Chun Wang*

Department of Rheumatology, China-Japan Friendship Hospital, Beijing, China.

Table S1. Clinical characteristics of DM patients and healthy controls.

|  | Gender | Age | Disease duration,yrs | ILD | Myositis specific antibodies* | Creatine kinase, levels | Muscle VAS scores | Muscle MHC-1 expression |
| --- | --- | --- | --- | --- | --- | --- | --- | --- |
| DM-1 | F | 44 | 0.25 | + | - | 4600 | 8 | + |
| DM-2 | F | 28 | 0.25 | + | - | 71 | 7 | + |
| DM-3 | F | 48 | 0.83 | + | - | 141 | 8 | + |
| DM-4 | F | 60 | 0.17 | + | - | 83 | 9 | + |
| DM-5 | F | 45 | 0.5 | + | - | 131 | 7 | + |
| DM-6 | F | 41 | 0.5 | - | - | 49 | 8 | + |
| DM-7 | F | 38 | 0.08 | - | - | 2485 | 9 | + |
| DM-8 | M | 34 | 0.5 | - | - | 328 | 7 | + |
| DM-9 | F | 46 | 0.25 | - | - | 140 | 6 | + |
| DM-10 | F | 45 | 0.33 | - | - | 1801 | 7 | + |
| DM-11 | M | 52 | 3 | + | Jo-1+ | 1510 | 9 | + |
| DM-12 | F | 67 | 0.17 | + | Jo-1+ | 59 | 8 | + |
| DM-13 | F | 49 | 3 | + | Jo-1+ | 424 | 7 | + |
| DM-14 | F | 32 | 0.67 | + | Jo-1+ | 5363 | 7 | + |
| DM-15 | F | 57 | 0.33 | + | Jo-1+ | 56 | 9 | + |
| HC-1 | F | 45 | NA | NA | NA | NA | NA | NA |
| HC-2 | M | 42 | NA | NA | NA | NA | NA | NA |
| HC-3 | F | 51 | NA | NA | NA | NA | NA | NA |
| HC-4 | F | 39 | NA | NA | NA | NA | NA | NA |
| HC-5 | F | 37 | NA | NA | NA | NA | NA | NA |

*: including anti-Mi-2, anti-Jo-1, anti-signal recognition particle, anti-threonyl-tRNA synthetase, anti-alanyl-tRNA synthetase, anti-glycyl-tRNA synthetase, anti-isoleucyl-tRNA synthetase antibodies, anti-MDA5, anti-TIF1γ, anti-NXP2, anti-SAE1, anti-HMGCR.

Table S2. LncRNAs and mRNAs detected by qRT-PCR and the primer sequences

| Gene | Sense | Sequence 5’-3’ |
| --- | --- | --- |
| ENST00000541196.1 | Forward | AAGTGTGCCCCCAAGTGATG |
|  | Reverse | GATGGCCAATAGTGGCTGGA |
| uc011ihb.2 | Forward | CAGGTCGCAGCCAAACATTC |
|  | Reverse | GTTGAGGCCAAAATCTCCGC |
| linc-DGCR6-1 | Forward | CTGTGCGAGACATTAGCCCT |
|  | Reverse | TACTGAAGTACCCCCTGCCA |
| ENST00000551761.1 | Forward | GGGCAGTTGGGAGACATCTT |
|  | Reverse | TCCTCCCATCCAATGCACAG |
| ENST00000583156.1 | Forward | CTGTACCGCGCATAGCAGAG |
|  | Reverse | CCTCTCACCAAGTGACACCG |
| USP18 | Forward | CGCTGTCATTTTCCATTTCCGT |
|  | Reverse | CTGAGGGGCCTCATGGTTAC |
| IFIH1 | Forward | TGCCAAGCATTACAAGAATAACCC |
|  | Reverse | TCTGGGGTCATATTGACGTGATG |
| FOS | Forward | GGAGGACCTTATCTGTGCGT |
|  | Reverse | TACACACTCCATGCGTTTTGC |
| ALDH3B2 | Forward | GGAGATCCACTACCCACCCT |
|  | Reverse | TTCTCTGTGTGACCCGTTGG |
| PFKFB3 | Forward | CAGCTGCCTGGACAAAACAT |
|  | Reverse | TCCGAGGCTGGACATTCCTA |
| GAPDH | Forward | TGTTGCCATCAATGACCCCTT |
|  | Reverse | CTCCACGACGTACTCAGCG |

Table S3. Top 10 upregulated and downregulated lncRNAs in dermatomyositis patients with interstitial lung disease (ILD) compared to patients without ILD.

| lncRNAs | Source database | Fold change | P value* |
| --- | --- | --- | --- |
| Upregulated |  |  |  |
| ENST00000428205.1 | ENSEMBL | 28.75 | 0.000018 |
| TCONS_00001982 | HumanLincRNACatalog | 14.46 | 0.000307 |
| XR_244292.1 | RefSeq | 14.25 | 0.00009 |
| TCONS_00023532 | HumanLincRNACatalog | 11.44 | 0.00219 |
| RNA95008|RNS_90_196 | CombinedLit | 9.82 | 0.00026 |
| ENST00000562866.1 | ENSEMBL | 8.67 | 0.000002 |
| ENST00000507566.1 | ENSEMBL | 8.36 | 0.00051 |
| TCONS_00028471 | HumanLincRNACatalog | 7.79 | 0.00016 |
| ENST00000595655.1 | ENSEMBL | 7.61 | 0.00021 |
| ENST00000512678.1 | ENSEMBL | 7.17 | 0.003 |
| Downregulated |  |  |  |
| ENST00000450016.1 | ENSEMBL | 101.03 | 0.000052 |
| ENST00000443162.1 | ENSEMBL | 100.02 | 0.0000002 |
| TCONS_00007244 | HumanLincRNACatalog | 16.08 | 0.00000016 |
| RNA143600 | Other ncRNAs^#^ | 13.87 | 0.0000026 |
| ENST00000592460.1 | ENSEMBL | 11.89 | 0.0000012 |
| RNA143598 | Other ncRNAs^#^ | 11.44 | 0.0000024 |
| TCONS_00019731 | HumanLincRNACatalog | 11.36 | 0.00000015 |
| TCONS_00009003 | HumanLincRNACatalog | 10.83 | 0.0000043 |
| RNA143588|rRNA_27_581 | Other ncRNAs^#^ | 10.65 | 0.000199 |
| ENST00000573982.1 | ENSEMBL | 10.55 | 0.002344 |

* The displayed P values have been adjusted by Benjamini-Hochberg correction.

^#^ Website: ftp://ftp.ensembl.org/pub/current/fasta/homo_sapiens/ncrna/Homo_sapiens.GRCh37.61.ncrna.fa.gz

Table S4. Top 10 upregulated and downregulated lncRNAs in dermatomyositis patients with anti-Jo-1 antibody compared to patients without anti-Jo-1 antibody.

| lncRNAs | Source database | Fold change | P value* |
| --- | --- | --- | --- |
| Upregulated |  |  |  |
| ENST00000587036.1 | ENSEMBL | 4.62 | 0.047 |
| uc031tge.1 | UCSC | 4.15 | 0.006 |
| TCONS_00009372 | HumanLincRNACatalog | 4.02 | 0.039 |
| TCONS_00022558 | HumanLincRNACatalog | 3.89 | 0.036 |
| ENST00000416104.1 | ENSEMBL | 3.69 | 0.017 |
| nc-HOXC8-148 | HOX Loci | 3.61 | 0.005 |
| uc010yfc.1 | UCSC | 3.45 | 0.012 |
| TCONS_00003800 | HumanLincRNACatalog | 3.32 | 0.013 |
| ENST00000585639.1 | ENSEMBL | 3.32 | 0.024 |
| XR_428946.1 | RefSeq | 3.11 | 0.034 |
| Downregulated |  |  |  |
| XR_110948.1 | RefSeq | 20.46 | 0.0005 |
| XR_110950.1 | RefSeq | 18.29 | 0.0005 |
| TCONS_00029753 | HumanLincRNACatalog | 16.39 | 0.0039 |
| ENST00000552784.1 | ENSEMBL | 8.08 | 0.0076 |
| ENST00000607613.1 | ENSEMBL | 6.07 | 0.0031 |
| ENST00000440778.1 | ENSEMBL | 4.78 | 0.0008 |
| uc031smp.1 | UCSC | 4.64 | 0.0029 |
| NR_108040.1 | RefSeq | 4.23 | 0.0111 |
| HIT000071394 | H-InvDB | 4.22 | 0.0438 |
| ENST00000435892.1 | ENSEMBL | 4.08 | 0.0097 |

* The displayed P values have been adjusted by Benjamini-Hochberg correction.

Table S5. Target gene prediction of differentially expressed lncRNAs.

| lncRNA-probe | lncRNA | predicted target mRNA | Correlation | p value |
| --- | --- | --- | --- | --- |
| p15332 | ENST00000450016.1 | XLOC_006069 | 0.999 | 1.5E-18 |
| p10267 | ENST00000416861.1 | XLOC_l2_007770 | 0.998 | 4.1E-11 |
| p14814 | ENST00000416100.1 | XLOC_006332 | 0.996 | 1.5E-18 |
| p28984 | ENST00000431700.1 | XLOC_l2_000399 | 0.996 | 1.3E-09 |
| p387 | ENST00000437865.1 | ANKRD20A2 | 0.996 | 1.7E-09 |
| p16602 | ENST00000415101.1 | XLOC_007832 | 0.995 | 1.5E-18 |
| p26746 | HIT000071394 | PLEKHA4 | 0.995 | 1.5E-18 |
| p9289 | ENST00000451884.1 | XLOC_l2_008203 | 0.995 | 1.5E-18 |
| p3758 | ENST00000552367.1 | DA381791 | 0.995 | 3.0E-09 |
| p33629 | linc-DGCR6-1 | USP18 | 0.993 | 1.4E-08 |
| p368 | ENST00000425010.1 | XLOC_000980 | 0.991 | 2.9E-08 |
| p12077 | ENST00000484765.2 | XLOC_002912 | 0.990 | 1.5E-18 |

**
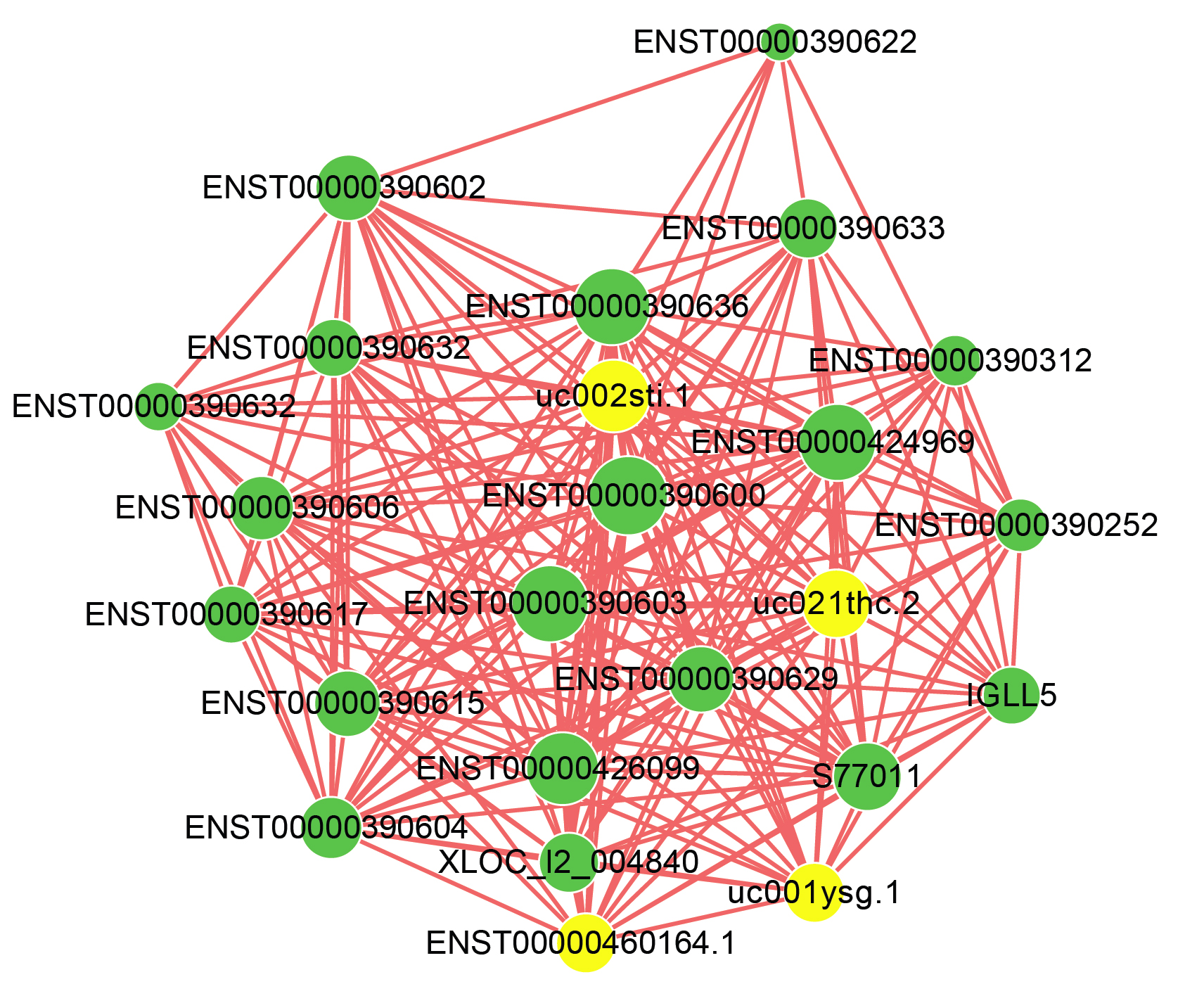
**

Figure S1. A representative mRNA-lncRNA co-expression network. In this network, yellow circular nodes represent lncRNAs and green circular nodes denote mRNAs. In total, the network involves 4 lncRNAs and 20 mRNAs.
